# Rapid and Progressive Loss of Multiple Retinal Cell Types in Cathepsin D-Deficient Mice—An Animal Model of CLN10 Disease †

**DOI:** 10.3390/cells10030696

**Published:** 2021-03-21

**Authors:** Mahmoud Bassal, Junling Liu, Wanda Jankowiak, Paul Saftig, Udo Bartsch

**Affiliations:** 1 Department of Ophthalmology, Experimental Ophthalmology, University Medical Center Hamburg-Eppendorf, 20246 Hamburg, Germany; mahmoudbassal@outlook.com (M.B.); junling.liu@stud.uke.uni-hamburg.de (J.L.); wandajankowiak@gmx.de (W.J.); 2 Institute of Biochemistry, Christian-Albrechts-University Kiel, 24118 Kiel, Germany; psaftig@biochem.uni-kiel.de

**Keywords:** bipolar cells, ganglion cells, lysosomal storage disorder, neuronal ceroid lipofuscinosis, photoreceptor cells, retinal degeneration, storage material

## Abstract

Vision loss is among the characteristic symptoms of neuronal ceroid lipofuscinosis (NCL), a fatal neurodegenerative lysosomal storage disorder. Here, we performed an in-depth analysis of retinal degeneration at the molecular and cellular levels in mice lacking the lysosomal aspartyl protease cathepsin D, an animal model of congenital CLN10 disease. We observed an early-onset accumulation of storage material as indicated by elevated levels of saposin D and subunit C of the mitochondrial ATP synthase. The accumulation of storage material was accompanied by reactive astrogliosis and microgliosis, elevated expression of the autophagy marker sequestosome 1/p62 and a dysregulated expression of several lysosomal proteins. The number of cone photoreceptor cells was reduced as early as at postnatal day 5. At the end stage of the disease, the outer nuclear layer was almost atrophied, and all cones were lost. A significant loss of rod and cone bipolar cells, amacrine cells and ganglion cells was found at advanced stages of the disease. Results demonstrate that cathepsin D deficiency results in an early-onset and rapidly progressing retinal dystrophy that involves all retinal cell types. Data of the present study will serve as a reference for studies aimed at developing treatments for retinal degeneration in CLN10 disease.

## 1. Introduction

Neuronal ceroid lipofuscinosis (NCL) comprises a group of mainly recessively inherited neurodegenerative lysosomal storage diseases usually starting in childhood [1,2,3]. While NCLs represent a genetically heterogeneous group of disorders, they are all characterized by the intracellular accumulation of autofluorescent storage material as a result of lysosomal dysfunction. The storage material, also termed ceroid, is composed of proteins, lipids, dolichols and metals [4]. Depending on the specific NCL form, the subunit c of mitochondrial ATP synthase (SCMAS) or sphingolipid activator proteins saposin A and D are major protein components of the storage material [4,5].

Originally classified mainly by the age at disease onset and ultrastructural characteristics of the storage material, NCLs are now grouped according to the affected gene [2,6,7]. To date, about 500 mutations (https://www.ucl.ac.uk/ncl-disease/mutation-and-patient-database/mutation-and-patient-datasheets-human-ncl-genes; accessed on 21 March 2021) in 13 different genes have been shown to cause different NCL forms (CLN1-8 and CLN10-CLN14) [7,8]. The identification of patients presenting with a NCL-like pathology but harboring no mutation in any of the currently known NCL-related genes suggests the presence of additional genetically distinct forms [9]. The majority of patients affected by lysosomal storage disorders (LSDs), including NCLs, present with clinical symptoms that are neuronal in origin, such as brain atrophy, seizures, cognitive deterioration and retinopathy [10,11].

CLN10 disease is caused by mutations in the gene encoding the lysosomal enzyme cathepsin D (CTSD), a ubiquitously expressed aspartyl protease [12,13]. To date, at least 12 different pathogenic mutations have been identified in the *CTSD* gene [14]. In addition to the critical role of the enzyme in the degradation of autophagic material in lysosomes, CTSD has been implicated in diverse other functions, including the activation and degradation of hormones and growth factors, activation of enzyme precursors, processing of brain antigens and regulation of apoptosis [15,16]. Loss-of-function mutations in the *CTSD* gene cause congenital NCL, the most severe NCL variant with prenatal disease onset and death within days or weeks after birth. Affected patients present with a severe phenotype, including skull and brain deformation, microcephaly, seizures and respiratory insufficiency [14,17,18,19]. Patients harbouring mutations in the *CTSD* gene resulting in decreased CTSD enzymatic activity, in comparison, present with an infantile or juvenile disease onset and a milder and more slowly progressing phenotype [20,21,22]. At the cellular level, pronounced neurodegeneration in CLN10 patients is accompanied by reactive astrogliosis, reactive microgliosis and demyelination. The inspection of brain biopsies and extraneural tissues, such as skin or muscle, revealed the presence of storage material displaying the ultrastructure of granular osmiophilic deposits (GRODs) [17,18,20,21].

Naturally occurring and transgenic animal models carrying mutations in the *Ctsd* gene display many phenotypic similarities to affected patients [23,24]. Similar to CLN10 patients, the severity of the phenotype correlates with the level of residual enzyme activity [25,26,27,28]. Cathepsin D knockout (*Ctsd* ko) mice are born viable and initially develop normally [25]. However, mutant mice start to accumulate SCMAS- and saposin D-containing storage material in different tissues early during development [29,30]. At 2 weeks of age, *Ctsd* ko mice display a progressive regression of visceral organs. In the brain, pronounced neurodegeneration becomes apparent during the third postnatal week and is accompanied by seizures, ataxia and bradykinesia. Reactive astrogliosis and microgliosis and progressive neuronal loss in different brain regions are accompanied by an accumulation of storage material with the ultrastructure of GRODs and fingerprint profiles [25,31,32,33]. The rapidly progressing phenotype of mutant mice ultimately results in premature death in the fourth postnatal week [25]. Together, findings demonstrate that the *Ctsd* ko mouse recapitulates many of the characteristic pathological features of congenital CLN10 disease.

Progressive visual impairment as a result of retinal degeneration is among the characteristic clinical symptoms of most NCLs [13,34]. Ophthalmic examinations of CLN10 patients with an infantile or juvenile disease onset revealed the deterioration of the retinal function and retinal structure with similarities to retinitis pigmentosa, as assessed by electroretinogram recordings and fundus examinations, respectively [20,21,22,35]. A follow-up examination of one of these patients revealed retinal atrophy at advanced stages of the disease, demonstrating the progressive nature of retinal degeneration in CLN10 [20]. Retinal degeneration is also among the pathological features of *Ctsd* ko mice [29,36]. Retinal degeneration in the mutant starts around postnatal day (P) 12, resulting in almost complete loss of photoreceptor cells at P25, shortly before the animal’s death [36]. Data on the progression of the retinal dystrophy, the different retinal cell types affected by the cathepsin D deficiency, and the molecular changes associated with the severe retinal pathology in this animal model are, however, limited. We, therefore, performed an in-depth analysis of the retinal phenotype of *Ctsd* ko mice, and quantified the thinning of different retinal layers, the progressive loss of various retinal cell types and the dysregulation of various lysosomal proteins. Results will serve as a reference for preclinical studies aimed at evaluating the efficacy of therapeutic strategies for the treatment of retinal degeneration in CLN10 disease.

## 2. Materials and Methods

### 2.1. Animals

Cathepsin D knockout (*Ctsd* ko) mice [25] on a C57BL/6J genetic background were obtained from heterozygous breeding pairs. Heterozygous mice displayed no pathological phenotype, in line with other studies [25,29]. We, therefore, used wild-type and heterozygous littermates as controls. Mice were genotyped using polymerase chain reaction and housed under standard conditions with ad libitum access to water and food in the animal facility at the University Medical Center Hamburg-Eppendorf (Hamburg, Germany).

### 2.2. Immunohistochemistry

*Ctsd* ko mice have a life expectancy of only around 26 days [25]. In the present study, we analysed retinas from 5-, 10-, 15-, 20-, 25-day-old *Ctsd* ko and age-matched wild-type or heterozygous mice of both sexes. Eyes were enucleated and fixed overnight in 4% paraformaldehyde (PA; Carl Roth GmbH, Karlsruhe, Germany) in phosphate buffered saline (PBS; pH 7.4). After cryoprotection with an ascending series of sucrose, eyes were frozen in Tissue-Tek (Sakura Finetek, Zouterwoude, The Netherlands) and serially sectioned with a cryostat (LEICA CM 1950, Leica Biosystems Nussloch GmbH, Nussloch, Germany) at a thickness of 25 µm, blocked in PBS (pH 7.4) containing 0.1% bovine serum albumin (BSA) and 0.3% Triton X-100 (both from Sigma-Aldrich Corp., St. Louis, MO, USA), incubated with primary antibodies (see Table 1) overnight, washed and incubated with appropriate Cy3-conjugated secondary antibodies (all diluted 1:200; all from Jackson Immunoresearch Laboratories, West Grove, PA, USA; research resource identifiers (RRIDs): donkey anti-rat Cy3: AB_2340666; donkey anti-rabbit Cy3: AB_2307443; donkey anti-goat Cy3: AB_2307351; donkey anti-sheep Cy3: AB_2315778; donkey anti-mouse Cy3: AB_2340813). Cone photoreceptor cells were labelled with biotinylated peanut agglutinin (PNA; 1:5000; Vector Laboratories, Burlingame, CA, USA) and Cy3-conjugated streptavidin (1:500; Jackson Immunoresearch Laboratories; RRID: AB_2337244). Preparations of retina flatmounts and immunostainings of retinal ganglion cells with antibodies to brain-specific homeobox/POU domain protein-3A (BRN-3A) were performed as described in [37,38]. Sections and flatmounts were stained with 4′, 6-diamidino-2-phenylindole (DAPI; Sigma-Aldrich) before mounting. Specificity of primary antibodies was controlled in immunoblot analyses of retina extracts and/or the localization and morphology of labelled cells. To control the specificity of secondary antibodies, incubation of some retina sections with primary antibodies was omitted in each experiment.

### 2.3. Retina Thickness Measurements and Cell Counting 

Optical sections with a thickness of 0.24 µm were taken from entire central retina sections using an AxioObserverZ.1 microscope equipped with an ApoTome.2 (Zeiss, Oberkochen, Germany) and ZEN2.3 software. The thickness of the entire retina, the outer retina (i.e., from the outer plexiform layer to the retinal pigment epithelium (RPE)) and the inner nuclear layer was measured at 18 equidistant positions between the nasal and temporal retina periphery using Fiji Image J 1.51 s software (Rasband, W.S., U.S. National Institutes of Health, Bethesda, MD, USA).

Rows of photoreceptor cell nuclei were counted in both the temporal and nasal retina close to the optic disc in sections stained with DAPI and antibodies to recoverin. BRN-3A-positive ganglion cells and calbindin-positive horizontal cells with a clearly visible nucleus were counted in entire retina sections, and their numbers per 1000 µm retina length were calculated. The density of ganglion cells in retinal flatmounts was determined as described in [37,38]. Numbers of arrestin-positive cone photoreceptor cells, protein kinase C alpha (PKCα)-positive rod bipolar cells and secretagogin (SCGN)-positive cone bipolar cells with a clearly visible nucleus were determined in three equidistant areas between the retina periphery and the optic disc in both the temporal and nasal retina, each with a width of 250 µm. PNA-labelled cone inner segments in P10 and older retinas were counted in the same areas, provided they were in direct contact with the outer nuclear layer to exclude obliquely oriented inner and outer segments from neighbouring optical sections from the analysis. In P5 retinas, all of the developing and very short PNA-positive inner and outer segments were considered. The density of ionized calcium-binding adapter molecule 1 (IBA1)-positive microglia cells was determined in the inner retina (i.e., from the inner nuclear layer to the vitreal margin) and the outer retina. All thickness measurements and cell counting were performed in a blinded manner on at least six animals of each genotype and age (i.e., 10-, 15-, 20- and 25-day-old animals) unless stated otherwise. Statistical analyses of cell numbers determined in retina sections were performed using the two-way ANOVA with “age” and “genotype” as between-group factors followed by a Bonferroni post hoc test using Prism 5.02 software (GraphPad Software, San Diego, CA, USA). Statistical analyses of ganglion cell numbers in retinal flatmounts and numbers of PNA-labelled cones in P5 retina sections were performed with an unpaired Student’s t-test. Retinal thickness measurements were analysed using a mixed three-way ANOVA with “genotype” and “age” as between-group factors and “distance from the optic nerve head” as within-group factor followed by a Bonferroni post hoc test for the interaction between “genotype” and “age” using Statistica 7 software (StatSoft, Inc., Tulsa, OK, USA).

## 3. Results

*Ctsd* ko mice display an early-onset and rapidly progressing phenotype characterized by lymphopenia, intestinal necrosis, loss of body weight, brain atrophy, motor impairment and retinal degeneration, and die around P26 [25,29,36]. To obtain detailed information on the progression of the retinal pathology, we analysed the retinal phenotype of this mouse model of CLN10 disease at the molecular and cellular levels.

### 3.1. Progressive Thinning of Ctsd ko Retinas

**Figure 1:**
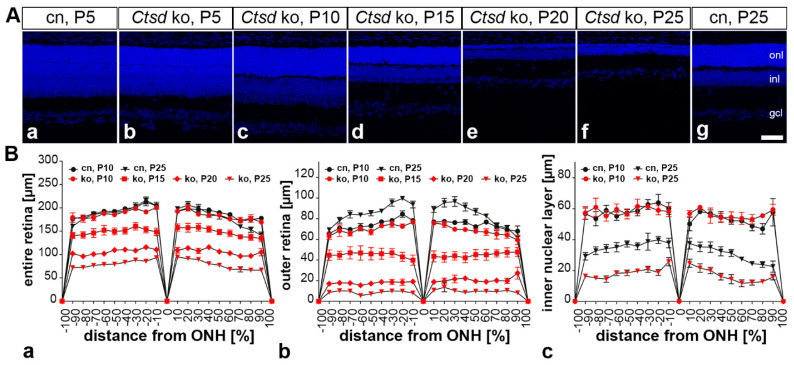
Progressive thinning of the *Ctsd* ko retina. (**A**) Retina sections from *Ctsd* ko mice at different ages (**b**–**f**) demonstrate a rapidly progressing retinal dystrophy in the mutant. Retinas from 5- (**a**) and 25-day-old control mice (**g**) are shown for comparison. (**B**) Quantitative analyses revealed no significant thinning of the entire retina (**Ba**), outer retina (**Bb**) or inner nuclear layer (**Bc**) in 10-day-old *Ctsd* ko mice when compared with age-matched control mice. Significant thinning of the entire retina (**Ba**), outer retina (**Bb**) and inner nuclear layer became apparent in 15-day-old *Ctsd* ko mice and further progressed with increasing age of the mutants (i.e., P20 and P25). For reasons of clarity, only data for selected ages are shown in (**B**). cn, control; gcl, ganglion cell layer; inl, inner nuclear layer; ko, knock-out; ONH, optic nerve head; onl, outer nuclear layer; P, postnatal day. Scale bar: 50 µm.

The inspection of central retina sections stained with DAPI revealed an apparently normal histology of the *Ctsd* ko retina at P5 and P10 (Figure 1Ab,Ac). A significant thinning of the *Ctsd* ko retina became evident at P15 (Figure 1Ad), and was pronounced at P20 and P25 (Figure 1Ae,Af, respectively). At the latter ages, the outer nuclear layer was almost completely atrophied when compared with age-matched control retinas (for a P25 control retina, see Figure 1Ag). We next determined the thickness of the entire retina, the outer retina (i.e., from the outer plexiform layer to the RPE) and the inner nuclear layer to quantify the progression of the retinal dystrophy in *Ctsd* ko mice, and to obtain hints whether retinal cell types other than photoreceptor cells are affected in the mutant retina (Figure 1B). There was a significant effect of the interaction among the “distance from optic nerve head”, “genotype” and “age” for the thickness measurements of the entire retina (F_51, 680_ = 2.04, *p* < 0.001) and the outer nuclear layer (F_51, 680_ = 3.65, *p* < 0.001), but not the inner nuclear layer (F_51, 680_ = 0.94, *p* > 0.05). Interactions between “genotype and age” were significant for all thickness measurements (entire retina: (F_3, 40_ = 58.02, *p* < 0.001); outer nuclear layer: (F_3, 40)_ = 94.17, *p* < 0.001); inner nuclear layer: (F_3, 40_ = 10.41, *p* < 0.001). The Bonferroni post hoc test revealed no significant differences in the thickness of the entire retina (Figure 1Ba), the outer nuclear layer (Figure 1Bb) or the inner nuclear layer (Figure 1Bc) between 10-day-old mutant and age-matched wild-type mice. However, the thickness of the entire retina, outer nuclear layer and inner nuclear layer was significantly decreased when compared to age-matched wild-type mice at P15 (*p* < 0.001), P20 (*p* < 0.001) and P25 (*p* < 0.001), as indicated by the Bonferroni post hoc test (Figure 1Ba–Bc).

### 3.2. Reactive Microgliosis and Astrogliosis

**Figure 2:**
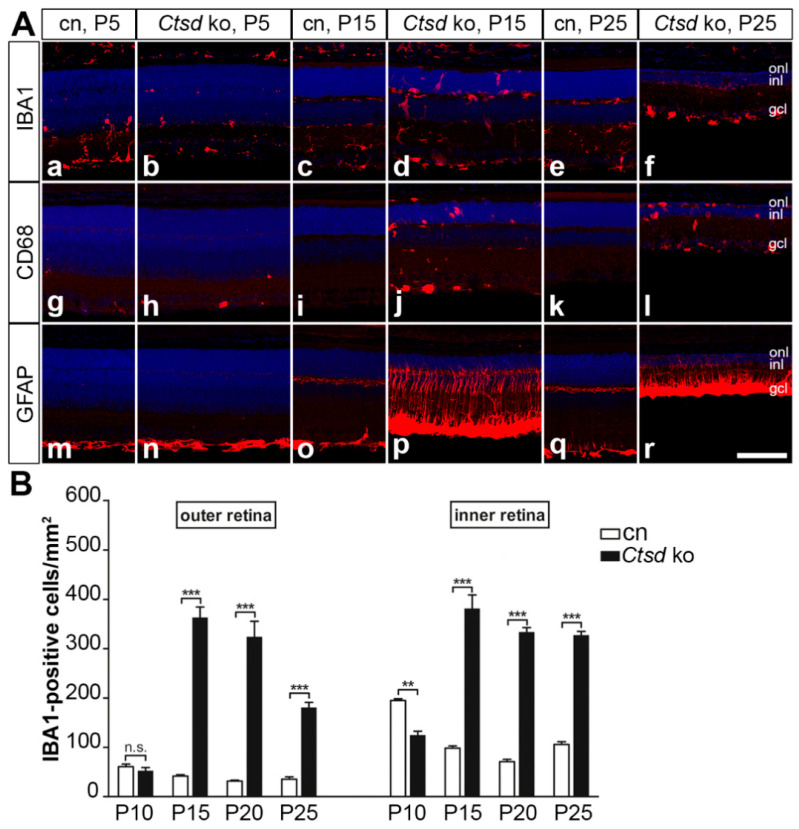
Reactive microgliosis and astrogliosis in *Ctsd* ko retinas. (**A**) At P5, IBA1-positive microglia cells were more numerous in control retinas (**Aa**) than in mutant retinas (**Ab**), while at P15 and P25, the density of IBA1-positive cells was markedly increased in mutant retinas (**Ad** and **Af**, respectively) when compared with age-matched control retinas (**Ac**,**Ae**, respectively). CD68-positive cells were confined to the inner retina of mutant mice at P5 (**Ah**), and increased in number and became additionally detectable in the outer nuclear layer and subretinal space of P15 (**Aj**) and P25 (**Al**). Control retinas were almost devoid of CD68-positive cells at all ages analysed (**Ag**,**Ai**,**Ak**). Expression of GFAP in control (**Am**,**Ao**,**Aq**) and P5 mutant retinas (**An**) was confined to retinal astrocytes. In P15 (**Ap**) and P25 (**Ar**) *Ctsd* ko retinas, expression levels of GFAP were markedly elevated in retinal astrocytes, and Müller cells were strongly GFAP-immunoreactive. (**B**) The density of IBA1-positive cells in the outer and inner retina of control (open bars) and *Ctsd* ko mice (filled bars) at different ages. Each bar represents the mean value (±SEM) of 6 animals. n.s., not significant; **, *p* < 0.01; ***, *p* < 0.001, two-way ANOVA. CD68, cluster of differentiation 68; cn, control; gcl, ganglion cell layer; GFAP, glial fibrillary acidic protein; IBA1, ionized calcium-binding adapter molecule 1; inl, inner nuclear layer; ko, knock-out; onl, outer nuclear layer; P, postnatal day. Scale bar: 100 µm.

To investigate the impact of CTSD deficiency on microglia cells/macrophages, retina sections were stained with anti-IBA1 and anti-cluster of differentiation 68 (CD68) antibodies. In the inner retina of 5-day-old control retinas (Figure 2Aa), the density of IBA1-positive cells was higher than in age-matched *Ctsd* ko retinas (Figure 2Ab). Quantitative analyses showed that the density of IBA1-positive cells in control mice was also increased in the inner retina, but not in the outer retina, at P10 (Figure 2B). At P15, we found a pronounced increase in the number of IBA1-positive cells in the mutant, with 362.7 ± 21.8 (mean ± SEM) positive cells/mm^2^ in the outer retina and 380.6 ± 28.4 positive cells/mm^2^ in the inner retina compared to 41.8 ± 2.5 and 98.2 ± 4.6 positive cells/mm^2^ in the outer and inner retina of the control mice, respectively (Figure 2Ac,Ad,B; *p* < 0.001 for both comparisons; two-way ANOVA). Similar numbers of IBA1-positive cells were found in the retinas of older *Ctsd* ko mice with the only exception of the outer retina, where the density of IBA1-positive cells decreased significantly between P20 and P25 (*p* < 0.001; Figure 2Ae,Af,B). CD68-positive microglia/macrophages became detectable in the inner retina of 5-day-old mutants (Figure 2Ah). In older mutants, CD68-positive cells were found throughout all retinal layers, including the outer nuclear layer, and were also frequently found in the subretinal space (for P15 and P25 *Ctsd* ko retinas, see Figure 2Aj,Al, respectively). CD68-positive cells were essentially absent from control retinas at all ages analysed (Figure 2Ag,Ai,Ak).

The expression of glial fibrillary acidic protein (GFAP) in the control retinas was confined to retinal astrocytes at all ages analysed (Figure 2Am,Ao,Aq). A similar expression pattern of GFAP was observed in 5-day-old mutant retinas (Figure 2An). In P10 *Ctsd* ko retinas, a few Müller cell processes were GFAP-positive, and GFAP expression in retinal astrocytes was elevated compared to the control retinas (Figure S1). In older *Ctsd* ko mice, the expression levels of GFAP in retinal astrocytes were massively increased, and Müller cells were strongly GFAP-immunoreactive (for 15- and 25-day-old retinas; see Figure 2Ap,Ar, respectively).

### 3.3. Accumulation of Storage Material and Dysregulation of Lysosomal Proteins

**Figure 3:**
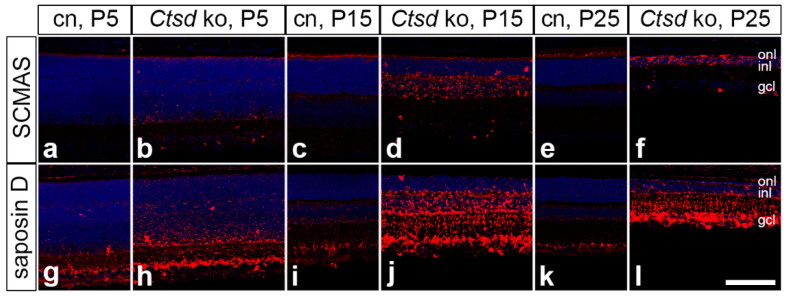
Accumulation of SCMAS and saposin D in *Ctsd* ko retinas. Levels of SCMAS and saposin D were moderately increased in P5 *Ctsd* ko retinas (**b**,**h**, respectively) when compared with age-matched control mice (**a**,**g**, respectively). A marked increase in SCMAS- and saposin D-immunoreactivity was observed in 15- (**d**,**j**, respectively) and 25-day-old mutant retinas (**f**,**l**, respectively) when compared to the corresponding control retinas (**c,e,i,k**). cn, control; gcl, ganglion cell layer; inl, inner nuclear layer; ko, knock-out; onl, outer nuclear layer; P, postnatal day; SCMAS, subunit c of mitochondrial ATP synthase. Scale bar: 100 µm.

To analyse the accumulation of storage material in *Ctsd* ko retinas, we performed immunostainings with antibodies to SCMAS and saposin D (Figure 3). The levels of both SCMAS (Figure 3b) and saposin D (Figure 3h) were elevated in *Ctsd* ko mice as early as at P5 when compared with the age-matched control retinas (Figure 3a,g, respectively), and further increased with the increasing age of the animals (Figure 3). SCMAS-immunoreactivity gradually shifted from inner retinal layers at P5 (Figure 3b) towards the outer retina at P15 (Figure 3d) and P25 (Figure 3f). The accumulation of saposin D also started preferentially in the inner retina and was particularly pronounced in the ganglion cell layer (Figure 3h). In older mutants, high levels of saposin D immunoreactivity were also observed in outer retinal layers (Figure 3j,l). Elevated levels of saposin D in mutant RPE cells were particularly evident at P25 (Figure S2). Compared to *Ctsd* ko retinas, levels of SCMAS- and saposin D-immunoreactivity in the control retinas were low at all ages analysed (Figure 3).

**Figure 4:**
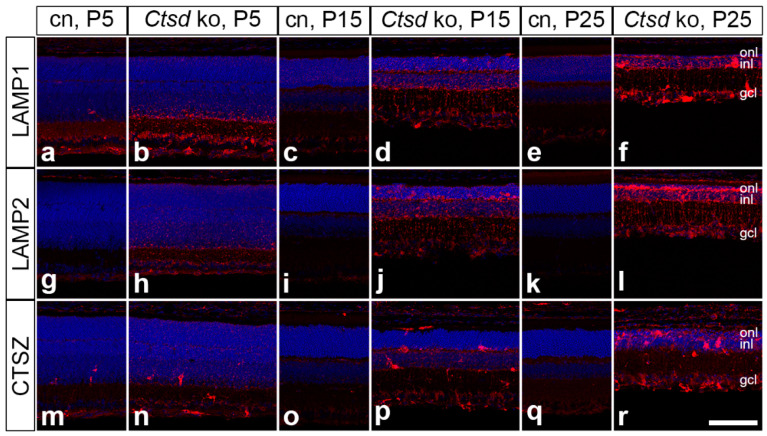
Dysregulation of lysosomal proteins in the *Ctsd* ko retina. LAMP1 (**b**), LAMP2 (**h**) and CTSZ (**n**) were moderately upregulated mainly in the ganglion cell layer and inner plexiform layer of P5 *Ctsd* ko mice when compared with age-matched control retinas (**a**,**g**,**m**, respectively). Expression levels of LAMP1, LAMP2 and CTSZ were further increased in P15 *Ctsd* ko retinas (**d**,**j**,**p**, respectively), and were prominent in all retinal layers of P25 mutant mice (**f**,**l**,**r**, respectively) when compared with the corresponding control retinas (**c,e,i,k,o,q**). Note the presence of strongly CTSZ-immunoreactive cells in mutant retinas at all ages analysed. cn, control; CTSZ, cathepsin X/Z/P; gcl, ganglion cell layer; inl, inner nuclear layer; ko, knock-out; LAMP1, lysosomal-associated membrane protein 1; LAMP2, lysosomal-associated membrane protein 2; onl, outer nuclear layer; P, postnatal day. Scale bar: 100 µm.

Next, we analysed the expression of the lysosomal proteins, lysosomal-associated membrane protein 1 (LAMP1) and lysosomal-associated membrane protein 2 (LAMP2; Figure 4). Elevated levels of LAMP1 and LAMP2 in *Ctsd* ko mice were evident as early as at P5 when compared with the control mice (compare Figure 4b with Figure 4a and Figure 4h with Figure 4g, respectively). At this age, prominent LAMP1 and LAMP2 expression was particularly evident in the inner retina. In older *Ctsd* ko retinas, increased expression levels of both proteins were also apparent in outer retinal layers (for 15- and 25-day-old *Ctsd* ko retinas; see Figure 4d,j and Figure 4f,l, respectively). In addition, there was a pronounced increase in LAMP2 in P25 *Ctsd* ko RPE cells (Figure S2). The expression levels of the lysosomal enzyme cathepsin X/Z/P (CTSZ) were also elevated in *Ctsd* ko retinas at P5, particularly in the inner retina (compare Figure 4m,n). As the retinal pathology advanced, levels of CTSZ further increased, and also became prominent in the outer retina (Figure 4p,r) and the RPE (Figure S2). Age-matched control retinas were almost devoid of CTSZ immunoreactivity (Figure 4o,q).

### 3.4. Expression of the Autophagy Marker SQSTM1/p62

**Figure 5:**
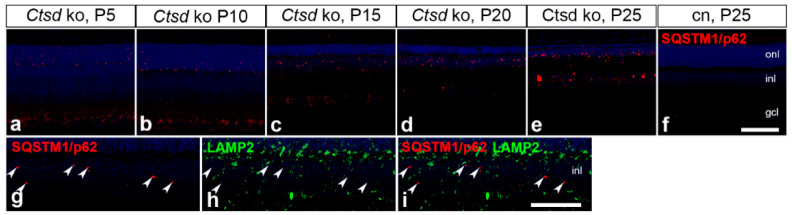
Expression of SQSTM1/p62 in *Ctsd* ko retinas. SQSTM1/p62-immunoreactive punctae in P5 (**a**) and P10 *Ctsd* ko retinas (**b**) were mainly restricted to the outer nuclear layer. In 15- (**c**), 20- (**d**) and 25- (**e**) day-old mutants, in comparison, expression of the autophagy marker was mainly confined to the inner nuclear layer. SQSTM1/p62-positive punctae were not detectable in retinas from control mice (for a P25 retina, see (**f**)). SQSTM1/p62 (**g**) and LAMP2 (**h**) showed hardly any co-localization (white arrowheads in **g**–**i**) in P15 *Ctsd* ko retinas. cn, control; gcl, ganglion cell layer; inl, inner nuclear layer; ko, knock-out; LAMP2, lysosomal-associated membrane protein 2; onl, outer nuclear layer; P, postnatal day; SQSTM1/p62, sequestosome 1/p62. Scale bar in (**f**): 100 µm; in (**i**): 50 µm.

In 5- and 10-day-old *Ctsd* ko retinas, sequestosome 1/p62 (SQSTM1/p62)-positive punctae were mainly confined to the outer nuclear layer (Figure 5a,b). In older *Ctsd* ko mice, in comparison, the expression of SQSTM1/p62 was mainly restricted to the inner nuclear layer (Figure 5c–e), where immunoreactive punctae became progressively more prominent with the increasing age of the animals. At P25, SQSTM1/p62 became additionally detectable in the ganglion cell layer (Figure 5e). No SQSTM1/p62 expression was found in the control retinas at any age analysed (for a P25 control retina, see Figure 5f). Of note, double immunostainings with antibodies to SQSTM1/p62 and LAMP2 revealed negligible co-localization of both proteins (Figure 5g–i), suggesting the localization of SQSTM1/p62 in early autophagic vesicles which have not yet fused with lysosomes.

### 3.5. Progressive Loss of Multiple Retinal Cell Types

Morphometric analyses of *Ctsd* ko retinas revealed a pronounced and rapidly progressing thinning of all retinal layers (Figure 1), suggesting the degeneration of multiple retinal cell types in the mutant retina. We, therefore, used a panel of cell type-specific markers to identify the retinal cell types affected by the CTSD deficiency and determined the time course of their degeneration.

#### 3.5.1. Rod and Cone Photoreceptor Cells

**Figure 6:**
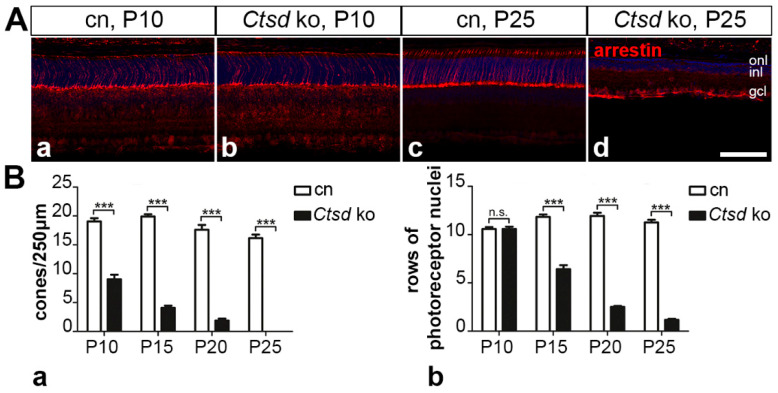
Degeneration of cone and rod photoreceptor cells. (**A**) The density of arrestin-positive cone photoreceptor cells was markedly reduced in *Ctsd* ko retinas at P10 (**Ab**) when compared with age-matched control retinas (**Aa**). At P25, mutant retinas were devoid of arrestin-positive cones (**Ad**; for a control retina, see **Ac**). (**B**) Quantitative analyses of mutant (filled bars) and control retinas (open bars) confirmed a significantly reduced density of cones in 10-day-old *Ctsd* ko mouse retinas, and a progressive loss of cones until P25 (**Ba**). The number of rows of photoreceptor nuclei was similar in *Ctsd* ko (filled bars) and control retinas (open bars) at P10, but then decreased rapidly in the mutant retinas with increasing age of the animals (**Bb**). Each bar represents the mean value (±SEM) of at least 6 animals. n.s., not significant; ***, *p* < 0.001, two-way ANOVA. cn, control; gcl, ganglion cell layer; inl, inner nuclear layer; ko, knock-out; n.s., not significant; onl, outer nuclear layer; P, postnatal day. Scale bar: 100 µm.

In the mouse retina, cone photoreceptor cells comprise only about 3% of all photoreceptors, while the rest are rod photoreceptors [40]. To analyse whether cones and rods are differentially affected in the mutant, we stained retina sections with antibodies to cone-arrestin or with the lectin peanut agglutinin to specifically visualize cones. Remarkably, PNA staining revealed a significantly reduced number of cones in *Ctsd* ko retinas already at P5, with 29.8 ± 3.5 (mean ± SEM) cones/250 µm retina length in mutant retinas compared to 42.4 ± 3.4 cones/250 µm in age-matched control retinas (*p* < 0.05, Student’s *t*-test; Figure S3). The determination of cone numbers in P5 retinas with anti-cone-arrestin antibodies was impossible due to the weak expression of the antigen at this early age. However, immunostainings of 10-day-old *Ctsd* ko retinas with anti-cone-arrestin antibodies confirmed a reduced density of cones (Figure 6Ab) when compared with age-matched control retinas (Figure 6Aa). Quantitative analyses revealed that the number of cones was reduced by more than 50% in the mutant at this age, with 9.0 ± 0.8 cones/250 µm retina length in *Ctsd* ko retinas as compared to 19.1 ± 0.6 cones/250 µm in control retinas (*p* < 0.001; two-way ANOVA; Figure 6Ba). Of note, *Ctsd* ko retinas were completely devoid of arrestin-positive cones at P25 (Figure 6Ad,Ba). Analyses of PNA-labelled retinas at P10 and P15 (Figure S4A) confirmed the data obtained with anti-arrestin antibodies. At P10 and P15, mutant retinas contained 8.4 ± 0.9 and 1.6 ± 0.4 PNA-labelled cones/250 µm retina length, respectively, while control retinas contained 17.8 ± 0.6 and 16.1 ± 0.5 PNA-labelled cones/250 µm, respectively (*p* < 0.001 for both comparisons; Figure S4B). In older retinas, the lectin produced a pronounced background, which made the reliable quantification of cones impossible.

Pronounced thinning of the outer nuclear layer (Figure 1A) additionally indicated a rapidly progressing loss of rod photoreceptors in the mutant. As rods comprise the vast majority of photoreceptor cells in the mouse retina, we determined the number of rows of photoreceptor nuclei at different ages to estimate the time course of rod degeneration. No significant difference in the number of photoreceptor nuclei was apparent between P10 mutant (10.6 ± 0.2 (mean ± SEM) rows) and control retinas (10.6 ± 0.2 rows; Figure 6Bb). However, in older mutants, the number of rows of photoreceptor nuclei decreased rapidly, with only 1.2 ± 0.1 rows remaining in mutants at P25 compared to 11.3 ± 0.3 rows in the age-matched control (*p* < 0.001, two-way ANOVA; Figure 6Bb). The morphological organization of the RPE as assessed by light microscopy was indistinguishable between the mutant and control mice until the terminal stage of the disease (Figure S2).

#### 3.5.2. Rod and Cone Bipolar Cells

**Figure 7:**
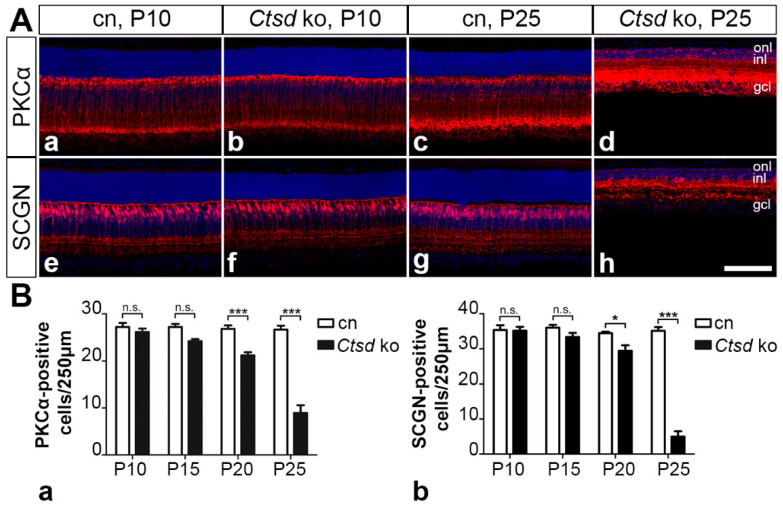
Degeneration of rod and cone bipolar cells. (**A**) The density of PKCα-positive rod bipolar cells and SCGN-positive cone bipolar cells in P10 *Ctsd* ko mice (**Ab**,**Af**, respectively) was similar to that in age-matched control mice (**Aa**,**Ae**, respectively). A pronounced loss of rod and cone bipolar cells was evident in 25-day-old mutant retinas (**Ad**,**Ah**, respectively) when compared with control retinas (**Ac**,**Ag**, respectively). (**B**) Quantitative analyses of mutant (filled bars) and control retinas (open bars) revealed a significant loss of rod (**Ba**) and cone bipolar cells (**Bb**) starting from P20. Each bar represents the mean value (±SEM) of 6 animals. n.s., not significant; *, *p* < 0.05; ***, *p* < 0.001, two-way ANOVA. cn, control; gcl, ganglion cell layer; inl, inner nuclear layer; ko, knock-out; onl, outer nuclear layer; P, postnatal day; PKCα, protein kinase C alpha; SCGN, secretagogin. Scale bar: 100 µm.

Significant thinning of the inner nuclear layer of mutant mice (Figure 1Bc) additionally suggested the degeneration of retinal interneurons. We, therefore, determined the number of PKCα-positive rod bipolar cells and SCGN-positive cone bipolar cells (Figure 7), and calbindin-positive horizontal cells (Figure S5) in the mutant and control retinas at different ages. At P10 and P15, we found similar numbers of rod and cone bipolar cells in the mutant and control retinas (Figure 7). However, the density of both cell types was significantly decreased in 20-day-old mutants, with 21.2 ± 0.6 (mean ± SEM) rod bipolar cells/250 µm retina length and 29.4 ± 1.6 cone bipolar cells/250 µm in *Ctsd* ko retinas compared to 26.8 ± 0.8 rod bipolar cells/250 µm and 34.5 ± 0.4 cone bipolar cells/250 µm in control retinas (*p* < 0.001 and *p* < 0.05, respectively, two-way ANOVA; Figure 7B). At P25, rod and cone bipolar cell numbers in mutant retinas accounted for only 33.5% and 14.1%, respectively, of the rod and cone bipolar cell numbers observed in the age-matched control retinas (Figure 7B). Different to bipolar cells, the number of calbindin-positive horizontal cells was not significantly altered in mutant retinas at P20 (Figure S5). At this age, *Ctsd* ko retinas contained 11.0 ± 0.8 (mean ± SEM) horizontal cells/mm retina length compared to 12.0 ± 0.5 horizontal cells/mm in control retinas. However, at P25, the density of horizontal cells was significantly reduced in the mutant (9.2 ± 0.4 horizontal cells/mm) when compared with the control mice (12.8 ± 0.6 horizontal cells/mm; *p* < 0.001, two-way ANOVA).

#### 3.5.3. Retinal Ganglion Cells

**Figure 8:**
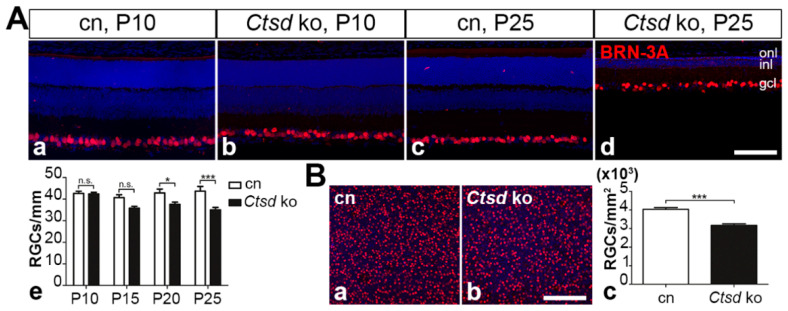
Degeneration of retinal ganglion cells. (**A**) Retina sections from *Ctsd* ko mice contained normal numbers of BRN-3A-positive ganglion cells at P10 (**Ab**) but reduced ganglion cell numbers at P25 (**Ad**) when compared with age-matched control retinas (**Aa**,**Ac**, respectively). Quantitative analyses of retina sections confirmed a significantly decreased RGC density in *Ctsd* ko retinas (filled bars in **Ae**) starting from P20 when compared with age-matched control retinas (open bars in **Ae**). n.s., not significant; *, *p* < 0.05; ***, *p* < 0.001, two-way ANOVA. (**B**) Analyses of retinal flatmounts from P25 *Ctsd* ko (**Bb**) and control mice (**Ba**) confirmed a significant loss of ganglion cells in the mutant at this age (**Bc**). ***, *p* < 0.001, Student’s *t*-test. Each bar in (**A**,**B**) represents the mean value (±SEM) of at least 6 animals. BRN-3A, brain-specific homeobox/POU domain protein 3A; cn, control; gcl, ganglion cell layer; inl, inner nuclear layer; ko, knock-out; onl, outer nuclear layer; P, postnatal day. Scale bars: 100 µm.

Finally, we analysed the impact of CTSD deficiency on retinal ganglion cell (RGC) survival using anti-BRN-3A antibodies. At P10 (Figure 8Aa,Ab) and P15, the control and mutant retinas contained similar numbers of ganglion cells (Figure 8Ae). However, starting from P20, there was a significant loss of ganglion cells of ~12% in *Ctsd* ko retinas (37.6 ± 1.1 (mean ± SEM) RGCs/mm retina length) when compared with the control retinas (42.8 ± 1.8 RGCs/mm; *p* < 0.05, two-way ANOVA; Figure 8Ae). At P25, the density of ganglion cells in the mutant was decreased by ~20% (35.0 ± 1.1 RGCs/mm) when compared with the control (43.7 ± 2.2 RGCs/mm; *p* < 0.001; compare Figure 8Ac–e). These findings were in line with results obtained from analyses of BRN-3A-stained retinal flatmounts from 25-day-old animals, where we found 3165.1 ± 84.9 (mean ±SEM) RGCs/mm^2^ in *Ctsd* ko retinas as compared to 4032.3 ± 88.6 RGCs/mm^2^ in control retinas, corresponding to a loss of 21.5% ganglion cells in *Ctsd* ko mice (*p* < 0.001; Student’s *t*-test; Figure 8B).

## 4. Discussion

CLN10 disease, the most severe NCL form, is caused by dysfunctions of the lysosomal aspartyl protease CTSD [17,20]. *Ctsd* ko mice [25] faithfully recapitulate many of the pathological features observed in CLN10 patients, including retinal degeneration [29,36]. In the present study, we performed a detailed analysis of the retinal dystrophy in *Ctsd* ko mice to obtain insights into the pathological alterations associated with CTSD deficiency. Specifically, we studied the molecular changes associated with retinal degeneration in this mutant mouse, identified the cell types affected in the *Ctsd* ko retina and quantified their degeneration during the course of the disease. A retinal pathology was already evident in young postnatal *Ctsd* ko mice, as indicated by an accumulation of storage material and a significant loss of cone photoreceptor cells. Reactive astrogliosis, reactive microgliosis and degeneration of rod photoreceptor cells became evident slightly later, while a significant loss of retinal interneurons and ganglion cells was observed at the end stage of the disease.

Neuroinflammation characterized by reactive microgliosis and reactive astrogliosis closely accompanies or even precedes neurodegeneration in different NCLs, and has been implicated in the progression of the neuropathology [41,42]. In the *Ctsd* ko retina, we found a few CD68-positive microglia/macrophages as early as at P5, and elevated levels of GFAP expression in retinal astrocytes and some Müller cells at P10. In older animals and coincident with the massive loss of photoreceptor cells, the density of microglial cells increased dramatically when compared with control mice, in line with a previous report [36]. Microglia had an amoeboid morphology indicative of an activated state. Of note, previous studies on the brain and retina of *Ctsd* ko mice have implicated activated microglia in the progression of neurodegeneration through the production of neurotoxic levels of nitric oxide. In support of this view, multiple intraperitoneal injections of nitric oxide inhibitors were shown to result in the significant attenuation of neuron loss in the thalamus and the inner retina of the mutant mouse [33,36,43]. A critical role of inflammatory immune responses in the progression of retinal dystrophies in NCL has also been demonstrated in mouse models of CLN1, CLN3 and CLN6 disease. Attenuation of the retinal pathology in these animal models was observed upon treatment with various immunomodulatory compounds, such as fingolimod, teriflunomide, minocyclin, curcumin or docosahexaenoic acid, and in genetic models with a compromised immune system [44,45,46,47,48].

The accumulation of storage material is a hallmark of all NCL forms, and was among the earliest pathological alterations observed in the developing *Ctsd* ko retina. The prevalence of two of the major components of lysosomal storage material, SCMAS and the sphingolipid activator proteins saposin A and D, differs between different NCL forms [5,13,34,49]. In the *Ctsd* ko retina, we detected elevated levels of SCMAS and saposin D as early as at P5. At this age, the accumulation of both proteins was mainly confined to the inner retina, from which they spread to the outer retina later during disease progression. The loss of retinal neurons, in comparison, was first apparent in the outer nuclear layer, subsequently in the inner nuclear layer and finally in the ganglion cell layer (see below). Thus, we observed no strict spatio-temporal correlation between the accumulation of storage material and neurodegeneration, similar to findings in the brain of a CLN6 sheep model [50,51]. Results from preclinical therapy experiments also suggest that the accumulation of storage material and neurodegeneration may not be causally linked. For instance, an AAV vector-mediated gene transfer of CTSD to the brain of *Ctsd* ko mice resulted in an attenuation of the neurological deficits and a pronounced extension of the mutant’s lifespan, despite the presence of high amounts of storage material in the brain [52]. Moreover, we recently showed that intravitreally injected human recombinant pro-CTSD significantly reduced the amount of storage material in the *Ctsd* ko retina without, however, attenuating retinal degeneration in the treated animals [53].

Similar to our observations in the dystrophic retinas of CLN1, CLN6 and CLN7 mouse models [54,55,56], we found an early and pronounced upregulation of lysosomal biogenesis in *Ctsd* ko retinas, as indicated by the increased expression levels of LAMP1, LAMP2 and the lysosomal cysteine protease CTSX/P/Z. The elevated expression of lysosomal proteins followed a spatiotemporal pattern closely resembling that of storage material accumulation, suggesting a link to lysosomal stress and activation of the transcription factor EB (TFEB) [57,58,59]. Impaired autophagy is a pathological hallmark of many lysosomal storage disorders [60,61,62], including CLN10 disease [53,63]. Accordingly, we found a striking accumulation of SQSTM1/p62, a receptor for autophagic cargo involved in the degradation of ubiquitinated proteins via the autophagosome-lysosome pathway [64,65]. SQSTM1/p62-positive aggregates were already apparent at P5, indicating early autophagic dysfunction in the mutant retina. In older animals, aggregates became detectable in a pattern that was similar to, but slightly preceded, that of retinal cell death. The lack of colocalization with LAMP2 suggested the impaired fusion of autophagosomes and lysosomes [66].

Morphometric analyses revealed a progressive thinning of the *Ctsd* ko retina starting at P15. Retina thinning was mainly the result of a rapidly progressing atrophy of the outer nuclear layer, in agreement with another report [36]. However, the thinning of other retinal layers was also observed. To identify the different cell types that are affected in the mutant retina and to quantify the progression of their degeneration, we analysed the retinal dystrophy at cellular level.

Of note, we found a significantly reduced density of cone photoreceptors in *Ctsd* ko retinas as early as at P5, slightly before a pronounced reactive microgliosis or astrogliosis became evident. In the mouse retina, cones are generated prenatally [67,68], suggesting that cones in the *Ctsd* ko retina start to degenerate shortly after they are born. However, it is also possible that the reduced number of cones is the result of a developmental defect. In older mutants, the loss of cones progressed rapidly, with no cones remaining at P25. Cones constitute only 3% of the total photoreceptor population [40]. The majority of photoreceptors are rods, which started to degenerate at P15. Similar to cones, the degeneration of rods progressed rapidly resulting in the loss of 90% of rods at P25. Together, the results demonstrate that the *Ctsd* ko mouse suffers from a cone-rod dystrophy. Early-onset photoreceptor degeneration has also been observed in mouse models of other NCLs, such as CLN4 [69], CLN5 [70], CLN6 [47,54,71], CLN7 [55] and CLN8 [72,73,74], indicating that photoreceptors are particularly sensitive to lysosomal dysfunctions. The identification of a few CLN3 and CLN7 patients presenting with visual impairment due to the loss of photoreceptors but without other neurological symptoms characteristic for these disorders is in line with this view [75,76,77,78,79,80,81]. Retinal pigment epithelial cells are essential for photoreceptor survival and function [82]. Disruption of the autophagy-lysosomal pathway and dysfunction of these phagocytically active cells may thus provide an explanation for the frequent involvement of photoreceptor cells in lysosomal storage disorders. While several studies have demonstrated the morphological, molecular and/or functional defects of RPE cells in different NCL forms, the precise impact of these pathological alterations on photoreceptor cell survival in these conditions is unknown [70,83,84,85,86,87]. The most prominent pathological alterations of Ctsd ko RPE cells found in the present study were significantly elevated levels of LAMP2 and saposin D, while the morphological organization of the retinal pigment epithelium, as assessed by light microscopy, was indistinguishable between the mutant and control mice until the terminal stages of the disease.

Another prominent feature of the retinal pathology of *Ctsd* ko mice was a significant thinning of the inner nuclear layer, although it was not as pronounced as that observed for the outer nuclear layer. At the cellular level, the thinning of this layer was mainly the result of a progressive degeneration of rod and cone bipolar cells. The loss of rod and bipolar cells was moderate at P20 (~21% and ~15%, respectively), but pronounced at P25 (~66% and ~86%, respectively). At the end stage of the disease, we additionally observed a significantly reduced number of horizontal cells. The degeneration of retinal interneurons has also been observed in animal models of other NCL forms. In fact, in some NCL forms, the retinal pathology has been reported to start mainly in the inner retina with the outer retina being only mildly affected. In a canine CLN2 model and in murine CLN1 and CLN3 models, for example, the retinal phenotype is characterized by a thinning of the inner nuclear layer and a relative preservation of the outer nuclear layer, a significant neurodegeneration in the inner nuclear layer and/or reduced b/a wave ratios in electroretinogram recordings [56,84,88,89,90,91]. Furthermore, results from a recent study suggest that the loss of photoreceptor cells in the *Cln6^nclf^* mouse is caused by defects in bipolar cells, which, however, start to degenerate only after a significant number of photoreceptors is lost [92]. Finally, we found a moderate loss of about 12% RGCs in the *Ctsd* ko retina at P20, which increased to ~20% at P25. The accumulation of high amounts of storage material in these large projection neurons already at initial stages of the disease, but the onset of RGC degeneration at relatively late stages of the retinal pathology, has also been observed in animal models of other NCL forms [55,56,93,94]. The loss of RGCs might be a direct consequence of lysosomal dysfunction in these neurons, or might occur secondary to pathological alterations in visual target centres in the brain or the myelinated optic nerve and tract [44,95,96]. Of interest in this context is that the lack of CTSD resulted in reduced levels of proteolipid protein and myelin basic protein, impaired lipid homeostasis, delayed the maturation of oligodendrocytes and disrupted central nervous system myelination [97,98]. Together, data demonstrate a striking variability of the retinal pathologies at the structural and functional levels between different NCL forms and highlight the need for precise knowledge of the retinal pathology of each NCL form in order to develop effective treatments.

Results from preclinical studies suggest that enzyme replacement strategies represent promising treatment options for NCLs caused by the dysfunction of soluble lysosomal enzymes, such as palmitoyl-protein thioesterase 1 (PPT1) in CLN1 disease, tripeptidyl peptidase 1 (TPP1) in CLN2 disease or CTSD in CLN10 disease [8,99,100,101,102]. Of note, the significant attenuation of disease progression has also been observed in a recent clinical trial on CLN2 patients treated biweekly with intracerebroventricular injections of recombinant TPP1 [103]. Remarkable therapeutic outcomes have also been achieved in the *Ctsd* ko mouse, despite the rapid disease progression using an adeno-associated virus (AAV) vector-mediated gene transfer to the brain and/or the viscera [52,104]. Furthermore, we recently showed that injections of recombinant human pro-CTSD (rhCTSD) resulted in the partial correction of various pathological markers in the brain, the attenuation of the visceral pathology and a prolonged life span of treated mice [53]. In addition, we also showed that intravitreal injections of rhCTSD resulted in a partial correction of several pathological markers and the attenuation of reactive microgliosis in the retina. However, the treatment had no significant impact on the progression of retinal degeneration [53]. Results from the present study will serve as a reference for ongoing work aimed at establishing treatments for retinal degeneration in CLN10 disease using AAV- and cell-based enzyme replacement strategies. Of interest in this context is that CLN10 patients carrying mutations in the *CTSD* gene that impair, but not completely abolish, the enzymatic activity of CTSD present with an infantile or juvenile disease onset and progressive retinal degeneration [20,21,35].

## Figures and Tables

**Table 1:** Primary antibodies.

Antigen	Dilution	Company/Reference	Catalog Number	RRID
brain-specific homeobox/POU domain protein 3A (BRN-3A)	1:200	Santa Cruz Biotechnology Inc., Santa Cruz, CA, USA	Sc-31984	AB_2167511
calbindin	1:2000	Sigma-Aldrich, St. Louis, MO, USA	C 9848	AB_476894
cathepsin D (CTSD)	1:2000	Santa Cruz Biotechnology, Inc.	Sc-6486	AB_637896
cathepsin X/Z/P (CTSZ)	1:100	R&D Systems GmbH	AF1033	AB_2088116
cluster of differentiation 68 (CD68)	1:1000	Bio Rad Laboratories, Kidlington, UK	MCA1957	AB_322219
arrestin	1:5000	Millipore, Temecula, CA, USA	AB15282	AB_1163387
glial fibrillary acidic protein (GFAP)	1:500	Dako Cytomation GmbH, Hamburg, Germany	Z0334	AB_10013382
ionized calcium-binding adapter molecule 1 (IBA1)	1:500	Wako Chemicals GmbH, Neuss, Germany	019-19741	AB_839504
lysosomal-associated membrane protein 1 (LAMP1)	1:2000	Santa Cruz Biotechnology, Inc.	Sc-19992	AB_2134495
lysosomal-associated membrane protein 2 (LAMP2)	1:200	Developmental Studies Hybridoma Bank, lowa City, IA, USA	ABL93	AB_2134767
protein kinase C alpha (PKCα)	1:500	Santa Cruz Biotechnology, Inc.	Sc-208	AB_2168668
recoverin	1:3000	Milllipore, Temecula, CA, USA	AB5585	AB_2253622
saposin D	1:4000	Konrad Sandhoff, Bonn, Germany [39]	N/A	N/A
secretagogin (SCGN)	1:2000	BioVendor Research and Diagnostic Products	RD184120100	AB_2034062
sequestosome 1/p62 (SQSTM1/p62)	1:1000	Enzo Life Sciences GmbH, Lörrach, Germany	BML-PW9860	AB_2196009
subunit c of mitochondrial ATP synthase (SCMAS)	1:1000	Abcam, Cambridge, UK	Ab181243	N/A

N/A, not available; RRID, research resource identifier.

## Data Availability

The data presented in this study are available on request from the corresponding author.

## Supplementary Materials

The following are available online at https://www.mdpi.com/2073-4409/10/3/696/s1, Figure S1: Expression of GFAP in 10-day-old *Ctsd* ko retinas; Figure S2: The retinal pigment epithelium of the degenerated *Ctsd* ko retina; Figure S3: Reduced number of cone photoreceptor cells in 5 days old *Ctsd* ko mice; Figure S4: Degeneration of PNA-labelled cones; Figure S5: Degeneration of horizontal cells.
