# Supplementary material for: Rapid and Progressive Loss of Multiple Retinal Cell Types in Cathepsin D-Deficient Mice—An Animal Model of CLN10 Disease
Source: Cells. 2021 Mar 21;10(3):696. doi: 10.3390/cells10030696 (PMC8003850; doi:10.3390/cells10030696)
Supplement: Supplementary file 1 [file cells-10-00696-s001.pdf]

Supplementary Materials

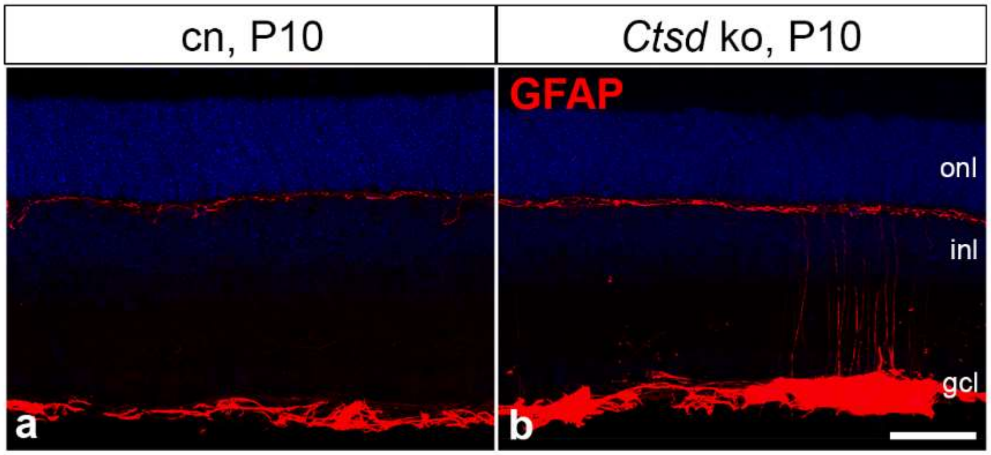

Figure S1. Expression of GFAP in 10-day-old *Ctsd* ko retinas. Expression of GFAP in retinal astrocytes was elevated in *Ctsd* ko retinas at P10 (b) when compared with age-matched control retinas (a). In addition, some Müller cell processes were GFAP-positive in mutant retinas at this age (b). cn, control; gcl, ganglion cell layer; GFAP, glial fibrillary acidic protein; inl, inner nuclear layer; ko, knock-out; onl, outer nuclear layer; P, postnatal day. Scale bar: 50 µm.

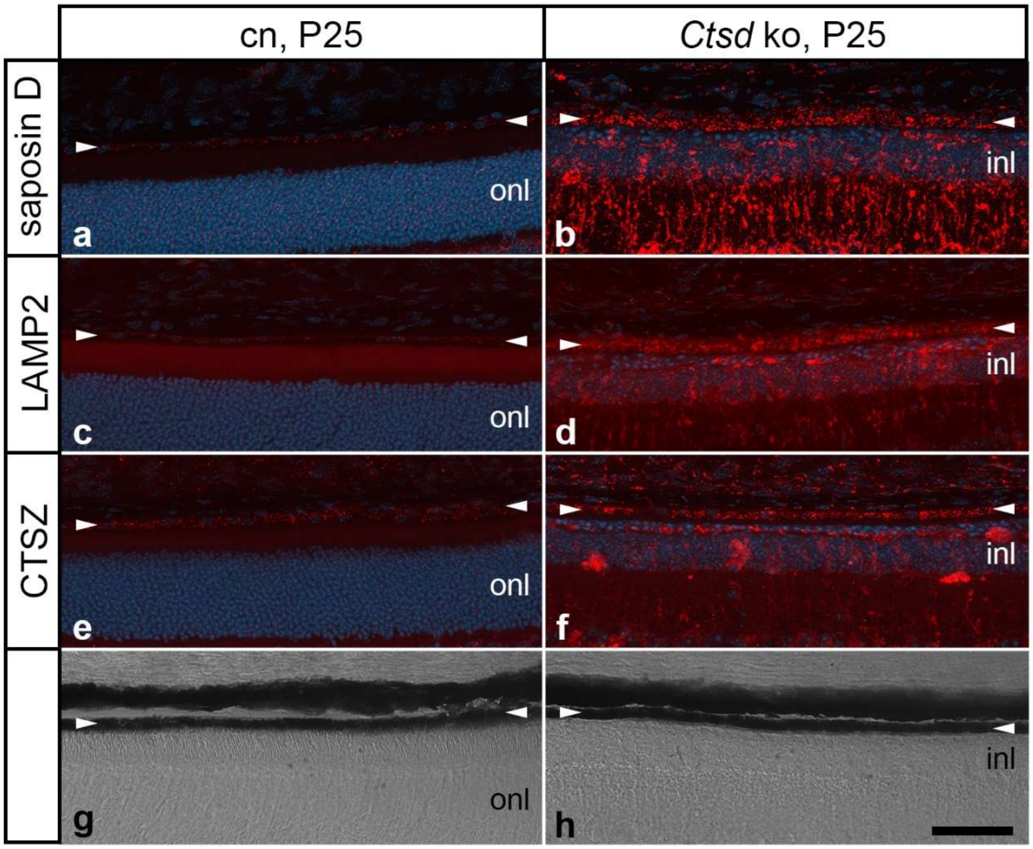

Figure S2. The retinal pigment epithelium of the degenerated *Ctsd* ko retina. Levels of saposin D (b), LAMP2 (d) and CTSZ (f) were significantly elevated in the RPE (marked with arrowheads in a-h) of *Ctsd* ko mice at P25 when compared with age-matched control animals (a, c and e, respectively). The morphological organization of the RPE at this age was indistinguishable between mutant (h) and control (g) retinas. cn, control; CTSZ, cathepsin X/Z/P; inl, inner nuclear layer; ko, knock-out; LAMP2, lysosomal-associated membrane protein 2; onl, outer nuclear layer; P, postnatal day. Scale bar: 50 µm.

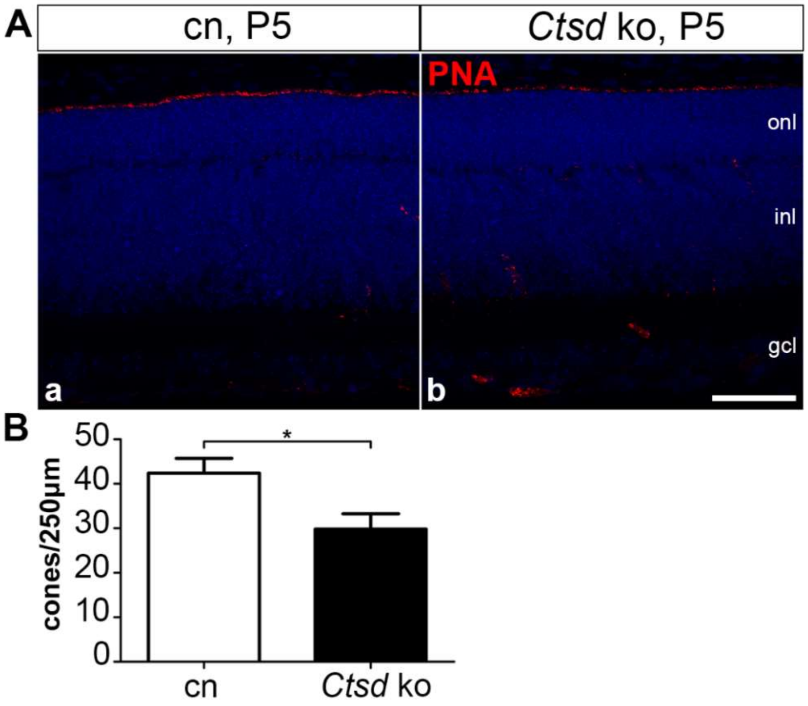

Figure S3. Reduced number of cone photoreceptor cells in 5 days old *Ctsd* ko mice. A) A markedly reduced number of PNA-labelled cones was detected in *Ctsd* ko retinas as early as at P5 (b) when compared to age-matched control retinas (a). B) Quantitative analyses confirmed a significantly reduced number of cones in the mutant retina. Each bar represents the mean value (±SEM) of 4 animals. *: $p<0.05$, Student´s t-test. cn, control; gcl, ganglion cell layer; inl, inner nuclear layer; ko, knock-out; onl, outer nuclear layer; P, postnatal day; PNA, peanut agglutinin. Scale bar: 50 µm.

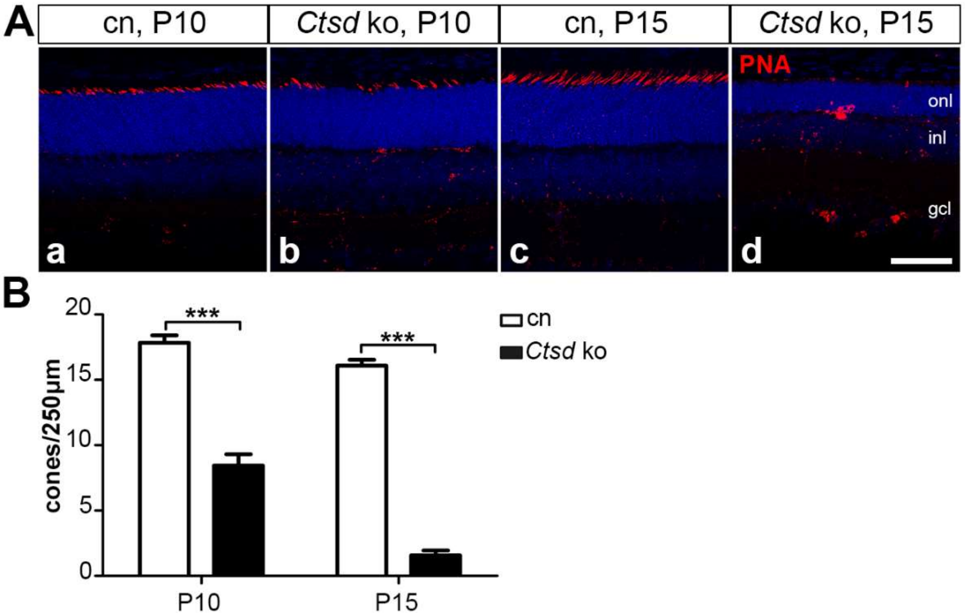

Figure S4. Degeneration of PNA-labelled cones. A) The number of PNA-labelled cones in *Ctsd* ko retinas was markedly reduced at P10 (Ab) when compared to control retinas (Aa). P15 mutant retinas were almost devoid of PNA-labelled cones (Ad). B) Quantitative analysis confirmed a pronounced and rapidly progressing loss of cones in mutant retinas. Each bar represents the mean value (±SEM) of 6 animals. ***, $p<0.001$, two-way ANOVA. cn, control; gcl, ganglion cell layer; inl, inner nuclear layer; ko, knock-out; onl, outer nuclear layer; P, postnatal day; PNA, peanut agglutinin. Scale bar: 100 µm.

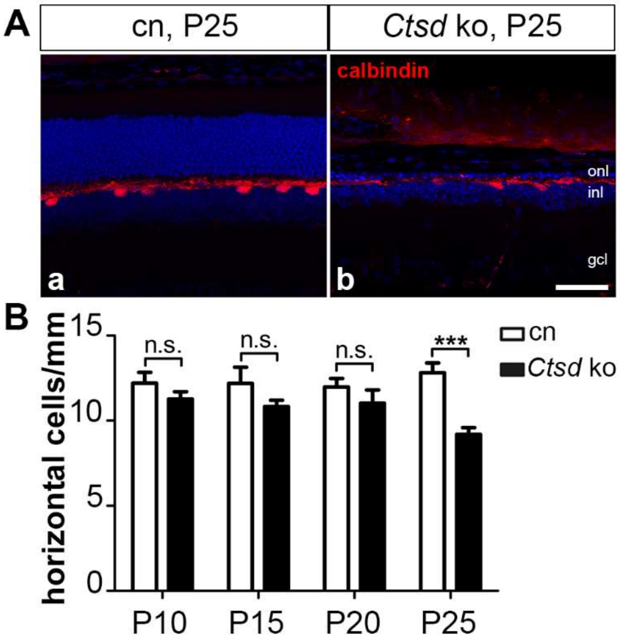

Figure S5. Degeneration of horizontal cells. A) The density of calbindin-positive horizontal cells was markedly decreased in *Ctsd* ko retinas at P25 when compared to age-matched control retinas. B) Quantitative analyses revealed that the number of horizontal cells was similar in mutant (filled bars) and control retinas (open bars) until P20, but significantly reduced in *Ctsd* ko retinas at P25. Each bar in (B) represents the mean value (±SEM) of 6 animals. n.s., not significant; ***, $p<0.001$, two-way ANOVA. cn, control; gcl, ganglion cell layer; inl, inner nuclear layer; ko, knock-out; onl, outer nuclear layer; P, postnatal day. Scale bar: 100 μm.
